# FluentDNA: Nucleotide Visualization of Whole Genomes, Annotations, and Alignments

**DOI:** 10.3389/fgene.2020.00292

**Published:** 2020-04-30

**Authors:** Josiah Seaman, Richard J. A. Buggs

**Affiliations:** ^1^Royal Botanic Gardens Kew, Jodrell Laboratory, Richmond, United Kingdom; ^2^School of Biological and Chemical Sciences, Queen Mary University of London, London, United Kingdom

**Keywords:** data visualization, nucleotide visualization, genome assembly, genome browser, chromosome structural variants, genome alignment, comparative genomics, space filling curves

## Abstract

Researchers seldom look at naked genome assemblies: instead the attributes of DNA sequences are mediated through statistics, annotations and high level summaries. Here we present software that visualizes the bare sequences of whole genome assemblies in a zoomable interface. This can assist in detection of chromosome architecture and contamination by the naked eye through changes in color patterns, in the absence of any other annotation. When available, annotations can be visualized alongside or on top of the naked sequence. Genome alignments can also be visualized, laying two genomes side by side in an alignment and highlighting their differences at nucleotide resolution. FluentDNA gives researchers direct visualization of whole genome assemblies, annotations and alignments, for quality control, hypothesis generation, and communicating results.

## Introduction

An intrinsic part of the analysis of genomic data is the summarization of large sequence datasets. This accomplishes three primary tasks: (1) quality checking an output, (2) understanding a sequence in context, and (3) communicating about sequence data in talks, posters and articles. This summarization is commonly achieved via metrics or by visualization. Simple metrics have the advantage of being precise, concise and easy to transmit, for example: N50, GC content, the mean size of exons and introns, and percent alignments. Tables of metrics can be used to convey information about, for example, overrepresented k-mers, or the location of low complexity regions or gene annotations. On the other hand, visualizations can give a broad, spatially explicit overview of sequence data.

Many software tools exist to visualize DNA sequence data, but in those that do include the bare sequence, it is only shown at the smallest scales. Genome browsers display nucleotide sequence only when zoomed to sub-kilobase scales, but not in broader overviews, and usually show annotations as linear blocks or line graphs in parallel tracks (Robinson et al., 2011; Kuhn et al., 2013; Buels et al., 2016). Multiple-Sequence Alignment (MSA) editors such as JalView have zoomable depictions of nucleotides or amino acids as colored blocks allowing variation between vertically organized samples to be picked out by the naked eye (Waterhouse et al., 2009; Katoh et al., 2017). Chromosome painting gives large scale summaries of genome structure, for example showing translocations between chromosomes using different colors (Serov et al., 2005; Kemkemer et al., 2006; Rasmussen et al., 2014). Circos plots visualize large scale rearrangements, such as syntenic blocks, with arcs (Krzywinski et al., 2009). SynTView uses heat-maps to depict variation among sequences (Lechat et al., 2013). To investigate tandem repeats and the subtle repeat pattern of codon bias, the tool SpectroFish uses a vertical axis to represent frequency (Sussillo et al., 2004; Sánchez and Lopez-Villasenor, 2006). DNA Walk visualizes sequence in terms of spatial steps (Arakawa et al., 2009). Ensembl, VisGenome and BugView all offer a browser view for aligned genomes, though they focus on larger features such as genes (Leader, 2004; Jakubowska et al., 2007; Zerbino et al., 2018) or gene presence/absence. These approaches do not show the negative space of intervening sequence (Hennig et al., 2015). In contrast, dot plots do show negative space and can handle densely connected or noisy data well; they are used for synteny analysis by duplicating the x-axis to form a square matrix of matching sequences (Lyons, 2008). More abstract visualizations which still use sequence are CGR, which shows k-mer representation (Deschavanne et al., 1999; Joseph and Sasikumar, 2006). BioJS (Yachdav et al., 2015), and Genome Projector (Arakawa et al., 2009) provide multiple ways of viewing genomic information and sequence variation at a range of scales. For genome assembly and pan-genome studies, visualization is used for quality control, for example, in Pan-Tetris (Hennig et al., 2015), Blobtools (Laetsch and Blaxter, 2017), Hawkeye and AMOS (Schatz et al., 2013).

In several areas of information technology, direct visualization of big data has accelerated data analysis. This has been key to the success of the company Palantir, whose software enables humans to work out complex interrelations in data (Khurana et al., 2009; Wright et al., 2009; Hossain et al., 2011). Other companies use the visualization of raw computer code in computer security research to seek the location of passwords, encryption, obfuscation and malware. One approach uses Hilbert space filling curves to calculate the entropy of programs (Conti et al., 2010; Cortesi, 2011). The software Cantor Dust uses this approach together with k-mer representation graphs^1^. Cantor Dust was acquired by Batelle, a think-tank for the CIA (Miller et al., 2001), though some features are available in open source derivatives Veles and Senseye ([Bibr B36][Bibr B43]).

Given the success of raw sequence visualization in other areas of big data analysis, it is reasonable to ask whether these techniques would also aid in genetic research and communication. A simple way to visualize large sequence files has been pioneered by DNA Rainbow (Bierkandt and Bierkandt, 2009), DNASkittle (Seaman and Sanford, 2009) and DDV (Neugebauer et al., 2015). These depict single DNA sequences as colored pixels (like an MSA editor), but introduce line breaks which wrap long sequences into 2D blocks. DNA Rainbow has a single raster column per chromosome with a fixed width of 3,500 pixels; this makes all but very large features difficult to discern by eye. DNASkittle has a variable column width optimized for tandem repeats and a suite of visualizations for exploring sequence similarity features in detail; this single column layout and 1D zoom is not ideal for use on large datasets, and it handles draft genomes and multiple chromosomes poorly. DDV introduces a more intuitive 2D zoom feature using sets of columns in a single layout, but does not support annotations.

In this paper, we present the tool FluentDNA, which visualizes sequence data with nucleotides as colors in a 2D layout with a zoomable interface. The layout can scale to accommodate any number of chromosomes and scaffolds. Individual nucleotides are visible when zoomed in and colors are averaged in zoomed out images. Even in the absence of any annotation of a genome, FluentDNA allows the human eye to pick out key features of a genome assembly by size and nucleotide composition. With practice, major features of chromosome architecture including centromeres, isochores, telomeres, and tandem repeats can be identified from the naked sequence because changes in k-mer usage cause changes in color and texture. Contamination is visible because of G/C content and coverage differences. FluentDNA expands on DDV’s visual paradigm with a suite of features such as the ability to handle multi-part FASTA files and whole genome assemblies, output different layout types, and visualize annotations, repeats, and alignments. It works on Windows, Mac and Linux. FluentDNA thus gives researchers direct visualization of their data files, for quality control, hypothesis generation, and communicating results. It can also promote the public understanding of science through public webpages and interactive museum displays.

## Methods

We designed our software to use the following conceptual methods for an easy-to-use whole genome visualization tool.

### Nucleotides as Pixels

Nucleotide sequences can be depicted as a series of pixels where the four bases are represented by four colors. The ideal color palette will conform to the following criteria: (1) high contrast; (2) friendliness to color-blindness; (3) typical nucleotide compositions should be viewable for over 20 min without causing discomfort (i.e. greens and blues should predominate) (Kaya and Epps, 2004; Mehta and Zhu, 2009).

### Depiction of One Dimensional Locality in Two Dimensions

To visualize long nucleotide sequences meaningfully in two dimensions, locality in the second dimension of the visualization must approximate locality in the one dimensional source data. The simplest way to do this is a linear sequence with frequent line breaks, ordered into a set of nested tiles. In this Tiled Layout, horizontally neighboring pixels are true neighbors in the source data, whereas vertical neighbors are spaced in the source data by the size of the column width. Another approach, referred to here as an Ideogram Layout, uses space filling curves. These are fractal shapes which fold a one dimensional continuous path to fill a 2D (or higher) area (Bially, 1969; Haverkort and van Walderveen, 2010) with no line breaks. One type, the Peano curve, is made of spirals of spirals, continually wrapping back in on itself to occupy the available space nearest to its origin. This process is recursive so locality is preserved at all scales. Peano curves approximate more closely the arrangement of nucleotide sequences in the interphase nucleus than do tiled arrangements (Lieberman-Aiden et al., 2009). However, it is impossible for the human eye to trace exact nucleotide sequences in a Peano curve: their utility is mainly restricted to broad overviews of data.

### Pan-and-Zoom Functionality

Eukaryotic genomes tend to be hundreds of megabases and even gigabases in length. When visualizing them in two dimensions, rapid and seamless pan-and-zoom functionality is essential. When zoomed out, pixel colors should be merged together to give an approximate representation of the nucleotide content by color.

### Mouseover Functionality

To move from visualization to analysis of specific genomic features with other software, users should be able to retrieve the sequence at any given point in the visualization simply by hovering over it. It should be possible to export snippets of DNA sequence as letter codes for further analysis.

### Annotations

Annotations can be visualized in two ways: (1) by directly highlighting nucleotides which are present in a genome feature; this works for both tiled and ideogram (see above) visualizations or (2) by a side-by-side column in a tile layout showing the location of features.

### Whole Genome Alignments

Whole genome alignments are commonly available as liftOver files. Using these, reference and query genome sequences can be visualized in side-by-side tile layouts where indels are depicted as gaps in one or the other genome. To highlight differences due to SNPs, indels, and rearrangements, extra columns can be added showing nucleotide differences between the two genomes, making them visible at a wide range of zoom scales. Different background colors can be used to indicate different types of rearrangements, though rearrangements within rearrangements will be hard to portray.

## Implementation

**FIGURE 1:**
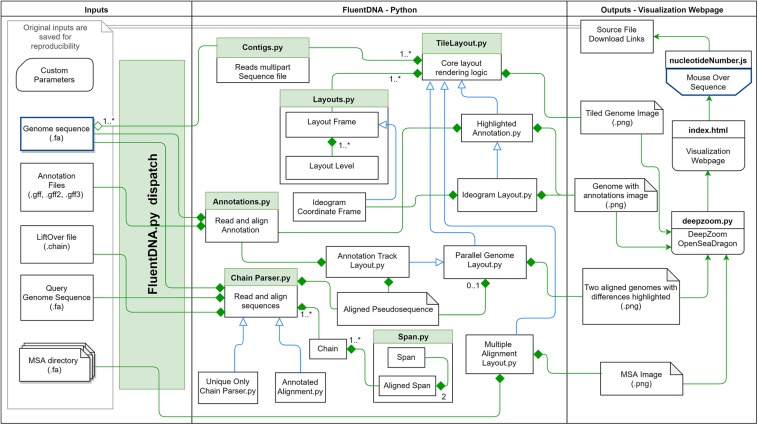
FluentDNA implementation UML showing the relationship between objects in the program. Green diamonds mean an object has one or more of the connected object. Blue arrow mean one object inherits all the properties of another object. Based on the input files provided (left), FluentDNA uses different rendering modes specified by the user, routed through FluentDNA.py. A FASTA sequence can be rendered in Tile or Ideogram style, each of which can also have gene annotations (GTF/GFF2/GFF3) overlaid with HighlightedAnnotation.py. Whole Genome Alignments handled by ChainParser.py require two FASTA files and a LiftOver file. Annotation Track Layout and Alignments both use the Parallel Genome Layout module and provide pseudosequences for further analysis. On the right, FluentDNA produces a web directory containing all input files and parameters (for reproducibility). The Visualization Webpage (top right) requires no installation and provides mouseover sequence retrieval. A glossary of files is listed in Supplementary Data Sheet S1.

These methodological concepts are implemented by FluentDNA in a Python code base with Javascript for browsing and mouseover. Python code handles the rendering of fasta files, annotations, and genome alignments as well as a file server. Javascript code depends on OpenSeadragon 2.4, Biojs Sequence 1.0, and jQuery 1.7 (Resig, 2006; Yachdav et al., 2015; OpenSeadragon, 2018). FluentDNA is available on MacOS and Windows as an executable command line tool or a GUI. It is available on all platforms as a python standalone library. The logical framework on FluentDNA is shown in Figure 1.

### Input Data

FluentDNA reads single or multiple sequence FASTA files of any size that the host machine’s memory can accommodate. For annotations, it reads GFF, GFF2, and GFF3 files. Visualizing whole genome alignments requires input of two genome assemblies in FASTA format and a liftOver file describing their alignment. The FluentDNA dispatch selects the appropriate layout based on input data and user parameters entered through the command line or GUI.

### Tile Layout

**FIGURE 2:**
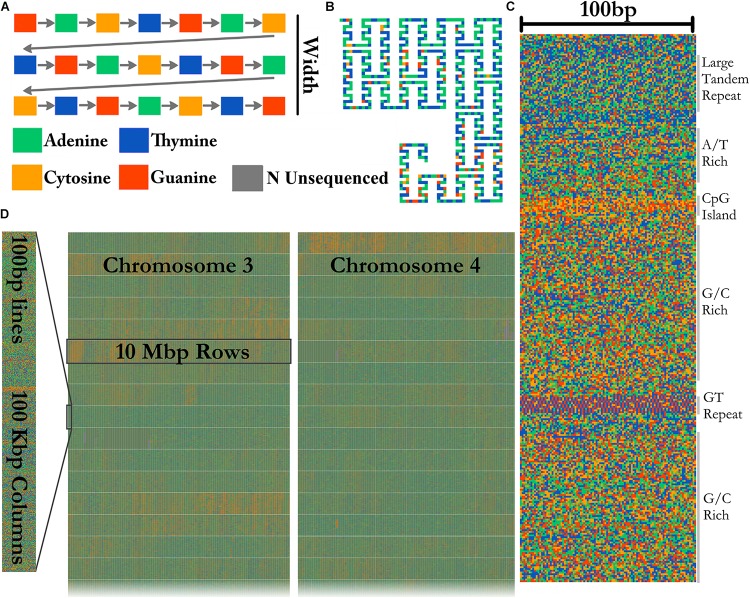
Visualization method. **(A)** In the Tile Layout sequence reads left to right like English text. **(B)** The Ideogram Layout uses Peano curves painted with sequence colors. In this example, the radices are x = (3,3,3), y = (5,3,3), and scale = 2 to insert whitespace around the curve. FluentDNA uses scale = 1, meaning the same path shape is present but there is no whitespace separating disjointed nucleotides. **(C)** The default width of one column is 100 bp. Features visible from bare sequence are annotated on the right. **(D)** In the default Tile Layout, 100 × 1000 bp columns are arranged in rows within mega-columns that represent chromosomes.

A FASTA file of any size can be visualized by FluentDNA in a tile layout (Figure 2). The default layout is arranged in powers of ten: rows of 100 pixels (each pixel representing one base), in columns of 1,000 rows containing 100 Kbp. One hundred columns are arranged in 10 Mbp mega-rows. Chromosomes occupy mega-columns composed of enough mega-rows to accommodate the largest chromosome (default 260 Mbp). Chromosomes are laid out side by side and several smaller chromosomes can share a single mega-column. In the default layout there is no white space within and between rows, 3 pixels of white space between columns, 9 between mega-rows, 700 pixels between chromosome columns. This default layout is defined in FluentDNA by a list of radices followed by a list of padding sizes: i.e., **[(100, 1000, 100, 26, 999**), (**0, 0, 3, 9, 700)]**. Users can change this using the **–custom_layout** option.

### Ideogram Layout

In Ideogram Layout, FluentDNA depicts the linear DNA sequence as a Peano curve (Figure 2B). This has an overall bounding box defining the 2D space filled by the curve, and internal bounding boxes that define how frequently the curve bends. The bounding boxes are defined internally by a set of (x, y) radices (Sagan, 1994).

### Pan and Zoom Functionality

The basic output of FluentDNA is a single master image file depicting the input DNA sequence. This file is inevitably very large for long sequences, making panning and zooming very memory intensive using direct image viewers. FluentDNA therefore automatically precomputes a “zoom stack” using the DeepZoom library, and sets up a local HTTP server which uses the OpenSeadragon platform (Khouri-Saba et al., 2013; OpenSeadragon, 2018) to view the zoom stack as a website using a web-browser. Interactive zooming can be disabled with the **–no_webpage** command line option. The position of the viewport, combined with the zoom level, generates a small list of tiles to be streamed to the browser. This allows for constant time performance on any device with any size dataset.

### Mouseover Algorithm

FluentDNA allows the selection of small sequence snippets in browser using mouse clicks over the image. Users can save 300 bp snippets of sequence using a keyboard shortcut which will add the coordinates and sequence to a log. This is often useful for BLAST or manually checking a result. Since the image is not itself a text object, FluentDNA uses an inverse function of each layout transformation to retrieve the original sequence position in the fasta input file and output the snippet’s DNA sequence in letter codes.

### Annotations

Annotation information from GTF, GFF2, or GFF3 files are visualized by FluentDNA as highlighted sequences within tiled or ideogram layouts, or in an annotation track next to a tiled layout. Currently, VCF and BED annotations are not supported.

Highlighted annotations are painted directly on top of the sequence using lightening, darkening, or outlines. Up to three different annotation files can be rendered with a different appearance. Gene annotations are specified with **–ref_annotation** and appear as lightened areas of the sequence, with lower opacity for introns and higher opacity for exons. Overlapping annotations are visible as doubly highlighted areas. Particular genes of interest can be highlighted with a drop shadow by specifying a second gene set with **–query_annotation**. Set intersections are used to detect shadows that collide with other annotated regions so that they can be adjusted to look natural. Repeat annotations specified with **–repeat_annotation** are rendered as dark regions. Gene name labels are rendered directly onto the rectangular bounding box of the annotated region. Label font size scales up for larger annotation areas. In the Tile Layout, gene labels are always placed at the start of the gene respective of strand: genes on the positive strand have their label at the top of the bounding box, while genes on the negative strand have labels at the bottom of the bounding box. In the Ideogram Layout, gene name labels are placed in the geometric centroid of the annotated nucleotides. The maximum and minimum x and y coordinates are used to determine a bounding rectangle to approximate the size of the gene region. Label font size and opacity is determined in a lookup table so larger genes get larger, more transparent labels painted onto them.

Annotations in a parallel track are depicted as a pseudosequence based on the GFF file. Only one annotation type can be present at any given location in the annotation track, so priority is given in order: CDS, exon, mRNA, gene. The annotation pseudosequence is interlaced side-by-side with the nucleotide sequence columns. As the annotation sequences are less information dense than the DNA sequence, the number of horizontal pixels in the annotation column can be set to a lower value than in the sequence column. The display width of the annotation column can be set with the **–annotation_width** parameter. When an annotation spans multiple columns, the median point is used to identify the column to fill with a label.

### Whole Genome Alignments

**FIGURE 3:**
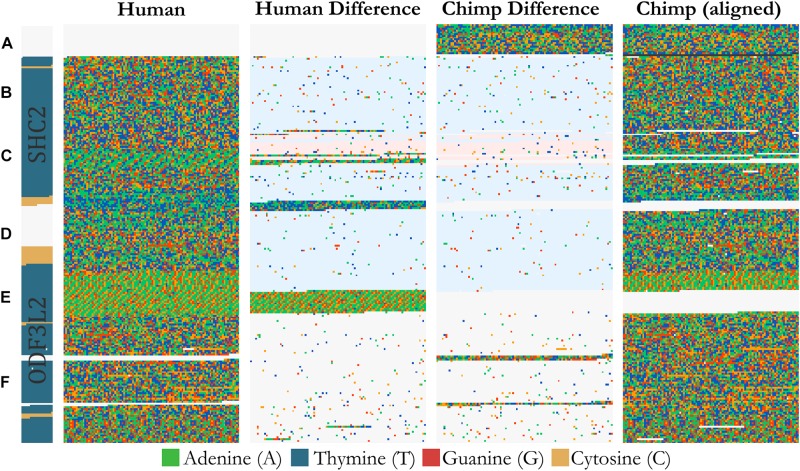
Design of an alignment visualization. An example from a whole genome alignment visualized in FluentDNA. 25 Kbp of Homo sapiens (Hg38) chr19:458,731 and Pan troglodytes (panTro5 2017). From left to right: gene annotation, Human sequence, Human unique sequence, Chimp unique sequence, and aligned Chimp sequence. Genome elements in the sequence can be seen without an annotation because of changes in nucleotide composition. Simple and tandem repeats appear as a texture. The two center “difference” columns generated by FluentDNA show the differences between the two sequences. Background colors indicate the source of the aligned region: syntenic (white), inversion and transposition (blue), or interchromosomal translocation (red). Two Human gene annotations for ODF3L2 and SHC2 appear on the left with blue introns and yellow exons. Letters mark biological features from top to bottom: Letters mark features visible from bare sequence: **(A)** Chimpanzee has a ∼1700 bp sequence not present in Human, **(B,D)** followed by an inversion, **(E)** which ends at a AAAC tandem repeat where Human has twice as many copies. Panel **(D)** contains a Human specific, highly A/T rich region that overlaps the end of SHC2’s exon annotation. **(C)** Inside the inversion, a simple AAAAGG repeat matches to sequence on another chromosome (red background). **(F)** Human and Chimpanzee share a syntenic region where Chimpanzee has 300 and 130 bp inserts.

FluentDNA can visualize whole genome alignments when provided with FASTA files for a reference and a query genome, and a liftOver file defining the genome coordinates of regions aligning between the two genomes. The liftOver file must have been previously generated using external whole genome alignment software. FluentDNA generates two gapped sequences from the reference and query genomes, using information from the liftOver file. It outputs a tiled layout with four columns: the reference genome, variants unique to the reference genome, variants unique to the query genome, and finally the query sequence (Figure 3). The two middle sequence columns highlight inversions, transpositions and translocations using background color (white: syntenic, blue: intrachromosomal transposition, red: interchromosomal translocation). In this way, differences between the two genomes in terms of SNVs, indels, inversions and translocations are visible at a range of zoom scales. FluentDNA also outputs a table quantifying these differences.

Whole genome alignment liftOver files, such as those available for many species pairs and assembly versions on the UCSC genome download site, contain a list of chain objects defined by a start position and strand in the reference and query genomes. Each chain is a series of entries with a contiguous alignment punctuated by gaps in the query or reference. Where two genomes are assembled to chromosomal level and highly similar, a single chain may cover much of the sequence data for each chromosome. Translocations and inversions introduce new chains. Multiple translocations from the same chromosome in the same orientation may be netted together depending on a distance cutoff. Ideally, a liftOver file will aggregate the alignment into as few chain objects as possible.

In order to turn the list of chains in a liftOver file into a visualization, it is necessary to linearize the alignment, pull in the sequence, and rearrange translocated sequences. FluentDNA sorts all chain entries in a UCSC Chained LiftOver file into a single list on the reference positive strand. The first large chain (referred to as the master chain) is used to establish a shared coordinate frame with the query genome. Other chains are then inserted into position, meaning all chains become intermixed. The reference genome stays in the same order and copy number but gaps may be inserted. The query genome sequence is rearranged to match the ordering and copy number of the reference genome (though if the liftOver file is for a reciprocal best alignment each sequence in the query genome will only be represented once). Each nucleotide index range tracks information about the source sequence: syntenic, intrachromosomal, or interchromosomal. New query sequence is brought in to fill unaligned gaps in the initial master chain alignment until all known alignments are composited into a single visualization.

When the master chain covers a large proportion of the query chromosome, unaligned query sequence is brought in with the master chain, introducing gaps in the reference and allowing the user to see sequence that is unique to the query genome. However, if the master chain covers only a small proportion of the query chromosome (for example because the genomes are highly divergent, or the query genome assembly is highly fragmented), then little to zero unaligned query sequence can be included in the visualization, and few gaps will be introduced into the reference genome. It will thus appear that the query genome is a subset of the reference genome, because regions of the query genome that cannot be aligned to the reference genome will not be placed within the visualization.

The background of the columns in the four-column alignment layout are colored to show which query alignments come from the master chain (shown by a white background: these are syntenic alignments), secondary chains with the same chromosome label as the master chain (shown by a blue background: these are normally due to inversions or translocations within a chromosome) and secondary chains with a different chromosome label (shown by a red background: these are normally due to translocations among chromosomes). FluentDNA can also output an image that only shows the nucleotides unique to the reference genome, using the option **–layout = unique**. The script **AlignmentStats.ipynb** can be used to aggregate genome alignment statistics for a whole genome.

### Phylogenomic Multiple Sequence Alignments

FluentDNA can visualize many multiple sequence alignments (MSA) in a single field of view, such as a set of genes aligned for a phylogenomic study. This allows users to, for example, pick out poorly aligned sequences. This function requires a directory of FASTA files as input. Each file in the directory contains multiple aligned sequences, representing one MSA. The file name is rendered as a text label over the sequence block. Files are either rendered in alphabetical order or in descending count of FASTA entries if **–sort_contigs** parameter is used. In the rendering engine, each MSA is listed as a separate layout with its own width and height. Mouseover sequence is handled by storing the origin point of each layout in the HTML.

### Image Generation for Publications

FluentDNA produces PNG visualizations at different scales for publications. The script **Image_resize_script.py** allows the user to set the level of magnification for any image output without introducing aliasing artifacts. Sequence images are approximately the same size as their FASTA files and do not compress, so FluentDNA does not use vector graphics.

### Publishing Results on Public Web Pages

Each genome visualized is stored in/results/inside of the FluentDNA installation folder. Visualization webpages can be published by placing this folder on any public facing server. No special FluentDNA server is required. For example, a visualization with **–outname** (=“HumanHg38”, the user would copy the folder results/HumanHg38 to the server then link to HumanHg38/index.html. Javascript runs on the client’s machine and downloads for all the source files are available through links to HumanHg38/sources/. Image browsing requires a small amount of traffic per user regardless of the size of the genome. Sequence mouseover generates more server traffic but can be disabled by deleting the/chunks/directory. Similarly, source downloads can be disabled by deleting the/sources/directory to protect private data.

### Museum Display

FluentDNA can support an interactive museum display allowing visitors to explore a whole genome assembly. A large poster is printed showing a tiled or ideogram image of a whole genome assembly, and overlaid with a touch sensitive screen. A flat screen monitor is built into the display. When visitors touch a point on the genome poster, a zoomed in image of that region is shown on the flat screen, together with annotation information and the DNA letter-code sequence. Detailed instructions for setting up such a display are given in Supplementary Data Sheet S2.

## Results

Here, we show the various outputs of FluentDNA for the latest version of the human genome, and its alignment to the chimpanzee genome. We also show how FluentDNA can be used to make a museum display. The commands to generate these visualizations are available in (Supplementary Data Sheet S3: Run scripts for Figures). The time and memory required to render specific figures is listed in Table 1.

**TABLE 1:** FluentDNA time and memory requirements.

	File Size	Time	Memory
HG chr18 Tile Layout without Annotation	80 Mbp	0:01:18	920 MB
HG chr18 Tile Layout with Annotation (Figure 4A)	80 Mbp	0:06:00	14 GB
HG chr18 Ideogram with Annotation (Figure 4B)	80 Mbp	0:13:16	18 GB
Hg38 whole genome without Annotation	3 Gbp	1:18:54	55 GB
Hg38 whole genome with Annotation (Figure 5)	3 Gbp	4:52:22	110 GB
Human-Chimp chr19 Alignment Visualization (Figure 3)	58 Mbp	00:08:00	18 GB

*Time performance is roughly linear with respect to input data size. A Highlighted Annotation takes three times as much memory as the nucleotide sequence alone since two images are created to make the overlay image. Compared to Tile Layout, Ideogram Layout takes additional time to compute the Peano curve coordinates.*

### Visual Analysis of the Human Genome

**FIGURE 4:**
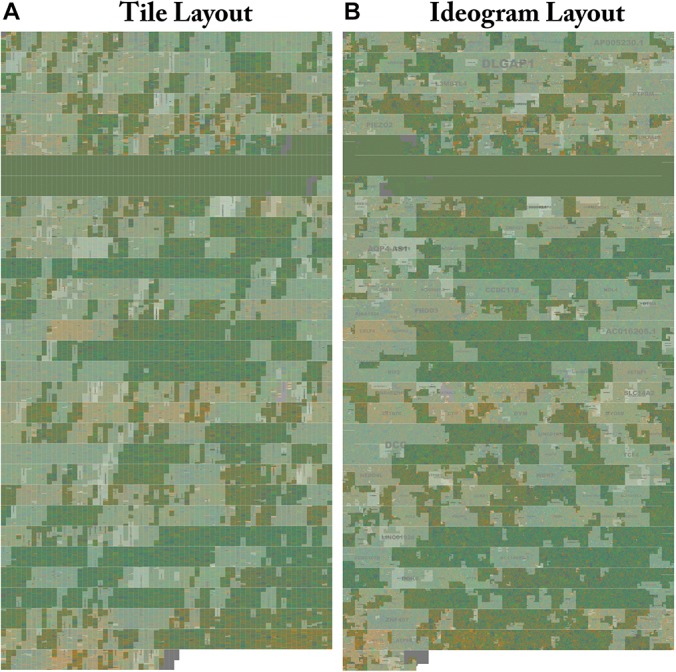
Side by side comparison of tiled and ideogram layouts. The same sequence is shown in two different layouts. Genes on Human Chr18 Tile and ideogram layouts side by side with highlighted annotation. **(A)** The whole structure of Human Chr18 with GenCode v30 Genes Highlighted rendered in the Tile layout. Gene labels are drawn in the center and scaled to the size of the region. Centromeres can be clearly seen as a two row region devoid of gene annotations. Isochores defined by changes in G/C content can be seen as changes in the background color. FluentDNA’s zooming interface allows users to see the whole chromosome then zoom in on areas of interest to see smaller features. **(B)** The whole structure of Human chromosome 18 is rendered in the Ideogram layout. The gene label DLGAP1 can be seen in the upper chromosome arm. Human gene annotations in Ideogram layout are designed to mimic the style of geographic maps with nation and city labels on different scales. The Peano curve snakes from left to right then right to left and is padded by a small amount of whitespace to mimic a chromatin fiber. Live versions: https://FluentDNA.com/Human_Genome_Hg38_chr18_with_Gen code_v30/ and https://FluentDNA.com/Human_Ideogram_Hg38_chr18_ with_Gencode_v30/.

**FIGURE 5:**
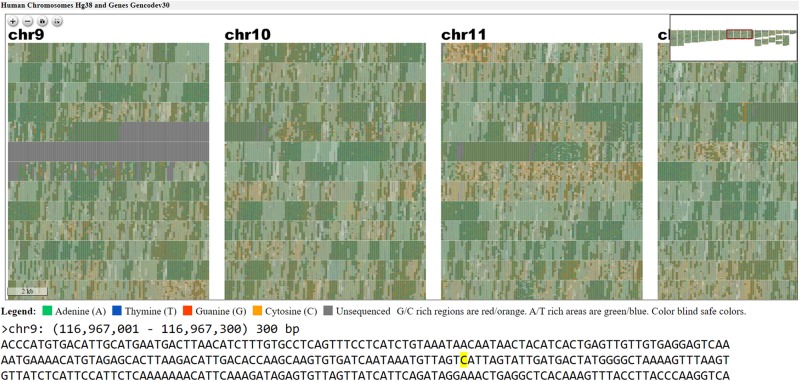
Webpage view of FluentDNA visualization of the entire human genome (hg38) in tiled layout with overlaid gene annotations. Users can zoom in on an element of interest to investigate in more detail. Users can see any sequence currently under the mouse pointer and save 300 bp snippets to a log including the scaffold name and position of each snippet. The exact nucleotide under the mouse is shown in a BioJS sequence component (Gómez et al., 2013). The live version with sequence retrieval alongside annotations is available: https://FluentDNA.com/Human_Genome_Hg38_and_Genes_Gencodev30/

A tile layout was generated for the Hg38 version human genome assembly chromosome 18 (Hg38) with default settings and highlighted gene annotations (Figure 4A). An ideogram (see Figure 4B) was made for the same chromosome with highlighted gene annotations for comparison. In both layouts the centromere is clearly visible as a homogenous gene free region. Individual genes are easier to pick out in the ideogram layout at the low level of magnification shown in Figure 4, as they have a more two-dimensional structure. At a high level of magnification the order of sequence is easier to read in the Tile Layout. At the whole genome level, users can see entire chromosome structures as well as prominent features (Figure 5).

**FIGURE 6:**
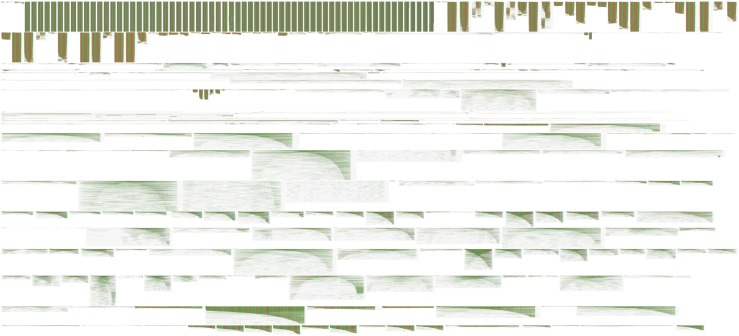
Multiple sequence alignment gallery visualization. This figure is a panoramic view of all instances of repeats on Human chr18 annotated by RepeatMasker. FluentDNA adjusts the layout width to match the consensus length of the repeat family. Starting in the Upper Left, major features are ALR centromere, Alu broken into subfamilies. Dominating the middle are long green repeats of L1, followed by the less conserved L2, then a collection of less abundant repeat families. RepeatMasker annotation positions downloaded from UCSC were used to extract the sequence for every non-simple repeat from Hg38, clustered by name, and aligned using only the repEnd coordinate (Kuhn et al., 2013; Smit et al., 2015). Live version: https://FluentDNA.com/Human_Hg38_Chromosome_18_Repeats_-_alphabetical/

In Figure 6, we use FluentDNA to visualize the repeat content of human chromosome 19 using a multiple sequence alignment gallery. RepeatMasker annotation positions downloaded from UCSC were used to extract the sequence for every non-simple repeat from Hg38, clustered by name, and aligned using the repEnd coordinate. This shows several families of LINES all with the same characteristic enrichment in 3′ ends. Alu repeats also have a distinctive di-mer structure where often only one L or R monomer is found in the genome. The result is equivalent to Figure 3 in Imbeault et al. (2017) which made it clear L1 has many more copies of the 3′ end than the 5′ end due to its copying mechanism.

### Human and Chimpanzee Comparison

We can also compare two chromosomes across species using Chain alignment files and the “Alignment” layout. Supplementary Figure S1 shows Human chr18 compared with the rest of the Chimpanzee genome, based on a liftOver file downloaded from UCSC. We can tell from the white background color in the central two columns that the entire lower chromosome arm is covered by a single syntenic alignment chain, indicating that Chimpanzee has an equivalent syntenic chromosome. The upper arm background color is blue, indicating the same chromosome, but not the master chain. This can be caused by an inversion in Chimp or, more likely, because the chaining algorithm has not joined chains from the upper and lower chromosome arms. Around the telomeres and centromeres we see smaller regions with a red background: this indicates these alignments are pulled in from other Chimpanzee chromosomes. These patches can be due to biological translocations or spurious alignments from another chromosome. Two obvious examples of this are in chunks 14,900,000 and 15,000,000 where regions brought in from other chromosomes show a markedly lower sequence identity in the middle difference columns. Finally, sequence unique to either the reference genome or query show up as interruptions in the four column layout when zoomed out. This allows users to quickly get a sense of how much the alignment covers and where. Users can zoom in on unique sequences of interest. For example, chunk 30,100,000 contains 50 Kbp of non-repetitive unique human sequence whereas the chunk before 30,000,000 contains 10 Kbp where the aligner simply failed to cover two regions which are visibly similar.

**FIGURE 7:**
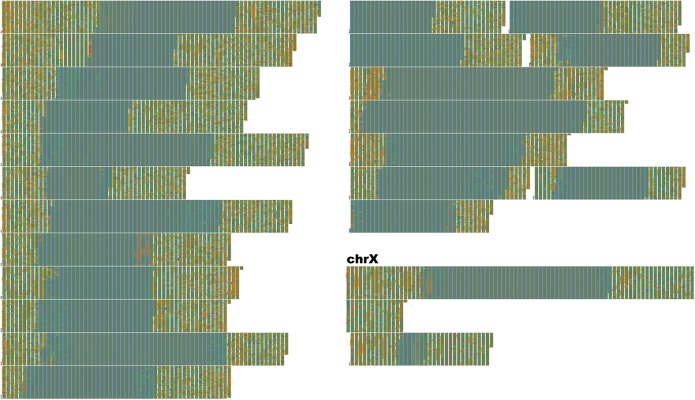
Human-unique sequence and annotation render of Hg38. FluentDNA’s Unique layout allows researchers to subtract one genome from another, leaving only the difference for inspection. Human sequence not covered by the alignment between Chimpanzee and Human is displayed in chromosome order within two layout pages. The unique portion is only 135 MBp, about the same size as chr9. Each visible row is a concatentation of the unique sequence from one chromosome starting with a small label, while chrX takes up more than one row and has a large label. This is because chrX has more Human unique sequence than any other chromosome. The gray-blue region in the middle of each chromosome is the sequenced centromere alongside a manual annotation (see section “Results” and Table 2). Outside the centromeres, a variety of tandem repeats and non-repeat content are visible. In the annotation track, introns are orange, exons are blue, and CDS is red. The visualization shows approximately half of the human-unique sequence is intronic, while exons are a small minority and CDS unique to Human are rarer still. Examples of Human specific protein sequence are shown in Table 3. Live version: https://FluentDNA.com/Unique_Human_Genes_and_Centromere_vs_Chimpanzee_PanTro6/

As an example of using FluentDNA for inspecting whole genome alignments, we used Human (Hg38, Dec. 2013) and Chimpanzee (PanTro6, Jan. 2018) assemblies available at UCSC and their corresponding liftOver file https://hgdownload.soe.ucsc.edu/goldenPath/hg38/liftOver/hg38ToPanTro6.over.chain.gz accessed March 2018. The full browsable alignment at nucleotide resolution is available at: https://FluentDNA.com/Human_Hg38_vs_Chimpanzee_PanTro6/. In addition to generating a visualization for each chromosome, FluentDNA calculates alignment statistics to quantify alignment coverage, sequence identity, and the distribution of gap sizes in the alignment (Table 2). Since centromeres and unsequenced regions correspond to biological features, calculations including N’s and centromeres are listed in parentheses. Initial alignment coverage is 95.57% (90.9%) of the Hg38 reference, and identity within the alignment of 98.65%. We used the “Unique” FluentDNA renderer to show the regions of Hg38 not covered by the alignment (Figure 7). This visualization immediately shows over half the unique Human sequence is centromere alpha satellite repeat, and sub-centromeric repeats, which are fully sequenced in Humans but represented by Ns in PanTro6. FluentDNA allows us to quantify these regions with more customizable precision than a generic repeat-masking would: by visual inspection, we were able to make a custom annotation of centromere and sub-centromeric regions in less than an hour. The start and end points we annotated for the centromeres can be seen in Supplementary Data Sheet S4. This allowed us to calculate the total Human unique sequence that is attributable to centromere repeats of all kinds. With centromeres excluded, coverage of the chimpanzee alignment is 97.9% of the human genome.

**TABLE 2:** Alignment Statistics for Human Genome Hg38 compared to the entire Chimpanzee PanTro6 genome.

Feature	Statistic
Reference Length (N’s included) (independent calc)	3,088,269,832
Reference length (No N’s) (independent)	2,937,639,113
Total alignment Length	2,807,378,393
Unaligned sequence within reference	149,173,906
Alignment length/Reference length	95.57%
Identical bases within alignment	2,769,610,997
Non-identical bases within alignment	37,767,396
Identical bases/Alignment length	98.65%
Number of gaps introduced in reference by alignment	2,139,409
Ref Gaps larger than 10 bp	241,691
Ref Gaps larger than 100 bp	54,321
Ref Gaps larger than 1000 bp	18,911
Ref N to query bp	150,636,009
Query N to ref in bp	15,139,025
Number of gaps introduced in query by alignment	2,216,928
Query Gaps larger than 10 bp	266,110
Query Gaps larger than 100 bp	53,587
Query Gaps larger than 1000 bp	17,145
Centromeric sequence length (manual annotation)	72,352,500
Reference length minus centromeres	2,865,286,613
Alignment length/Reference length minus centromeres	97.98%
Identical bases/Reference length minus centromeres	96.66%

*The top 18 rows are available for any alignment processed with FluentDNA. Using the script Stats_Aggregator.ipynb, which collects statistics across many chromosomes and aggregates them into a summary of a whole genome alignment. Statistics for chromosomal alignment are generated automatically as a single file per reference chromosome by FluentDNA. In the Hg38 versus PanTro6 alignment, “Alignment length/Reference length” is lower than expected because Hg38 has almost fully sequenced centromeres, whereas the PanTro6 has largely unassembled centromeres. Using FluentDNA’s Unique Only Chain Parser, we rendered the unalignable regions of Hg38 and used the tool to mark the beginning and end of sub-centromeric regions based on sequence. Using these coordinates, we calculated Alignment length/Reference length minus centromeres. Statistics for this manual analysis are shown in the final four rows.*

FluentDNA also quantifies the source of aligned sequence for every nucleotide, shown by different background colors in the visualizations (Using the script AlignmentStats.ipynb). In the UCSC whole genome alignment of PanTro6 to Hg38, the first chain for each chromosome covers 52.9% of Hg38 excluding Ns and centromeres (49.1% including Ns and centromeres) of the genome; 78.8% (73.1%) is covered by the first two chains and 87.3% (81.0%) is covered by the first three chains. In total, 95.7% (88.8%) of the Hg38 genome is covered by chains derived from the same homologous chromosome in chimpanzee (including chimpanzee chromosomes 2A and 2B as both corresponding to human chromosome 2). Another 2.2% (2%) of Hg38 is covered by chimpanzee chains that are not derived from homologous chromosomes for a total of 97.9% (90.8%). Full statistics for each chromosome can be found in Supplementary Table S1.

We can use the visualization to find and explore the context of putative human-specific protein coding sequences. Browsing the webpage for Figure 7, one can find rare patches of red in the annotation column, indicating protein coding (CDS) sequence. For example, we identified 8 segments on Chr1 containing unique CDS and used the FluentDNA feature to clip and store each of the sequences (Table 3). This log was then submitted as a BLAST query against Hg38 which returns annotated features. Gene functions returned include: amiloride-sensitive sodium channel subunit delta, vascular cell adhesion protein, neuroblastoma breakpoint family member 19, mucin-1 isoform 19 precursor, HHIP-like protein 2 precursor, olfactory receptor 2T10 (Table 3). HHIPL2^2^ is related to HOX genes, possibly crucial, and deserves closer scrutiny. This result is caused by a 200bp segment of protein coding DNA in Hg38 that is not covered by the PanTro6 alignment. BLAST searches for this sequence in *Pan troglodytes* returns hits at the expected 98.66% identity, so it is safe to conclude that the sequence is present but the whole genome alignment is imperfect. In contrast, olfactory receptor 2T10 is genuinely missing from *Pan troglodytes* but present in *Gorilla gorilla* and *Pongo abelii*.

**TABLE 3:** Example human exons on Hg38 chromosome 1 showing no alignment with PanTro6.

			
Start position in Ch1)	300 bp sequence from human CDS showing no chimpanzee alignment	BLAST Chimp	Gene Feature
			
122,005	GGCCCAGGGTAGGGAGGCCTGAGTGGGTGCAGGCCGGGCCCTGCTGAGGCCACTCTG CACACAGGCTGCAGCCCAGACGCCCCCCAGGCCGGGGCCACCATCAGCACCACCACC ACCACCCAAGGAGGGGCACCAGGAGGGGCTGGTGGAGCTGCCCGCCTCGTTCCGGGA GCTGCTCACCTTCTTCTGCACCAATGCCACCATCCACGGCGCCATCCGCCTGGTCTGCT CCCGCGGGAACCGCCTCAAGACGACGTCCTGGGGGCTGCTGTCCCTGGGAGCCCTGG TCGCGCTCTGCTG	present (79% cov.)	amiloride-sensitive sodium channel subunit delta
1,782,205	CAAGAATACAGTTATTTCTGTGAATCCATCCACAAAGCTGCAAGAAGGTGGCTCTGTGAC CATGACCTGTTCCAGCGAGGGTCTACCAGCTCCAGAGATTTTCTGGAGTAAGAAATTAGA TAATGGGAATCTACAGCACCTTTCTGGAAATGCAACTCTCACCTTAATTGCTATGAGGATG GAAGATTCTGGAATTTATGTGTGTGAAGGAGTTAATTTGATTGGGAAAAACAGAAAAGAGG TGGAATTAATTGTTCAAGGTGAGTAGAATGTGAAAAAGGAATGATAAAGGTGCTGTCA	missing	vascular cell adhesion protein 1 isoform b precursor
5,892,205	TGAAATCTAGCTGGGGCTGTGTGGTTTCTGATTCCCCCTGGCTTATTCTTTACTTTTTCCC ACTTTTCCAGGCTCAGCAGGGAGCTGCTGGATGAGAAAGGGCCTGAAGTCTTGCAGGA CTCACTGGATAGATGTTATTCAACTCCTTCAGGTTGTCTTGAACTGACTGACTCATGCCA GCCCTACAGAAGTGCCTTTTACATATTGGAGCAACAGTGTGTTGGCTTGGCTGTTGACAT GGATGGTGAGTACCTTTCTATGAAGGTGATAAGGATCCACTGAGTCTTCTGGTTAGGGTCA	present	neuroblastoma breakpoint family member 19
5,966,905	CTGTCCCCAGGTGGCAGCTGAACCTGAAGCTGGTTCCGTGGCCGGGGCCAGAGTGA CATCCTGTCCCTGAGTGGTGGAGGAGCCTGAACCGGGGCTGTGGCTGGAGAGTACGC TGCTGGTCATACTCACAGCATTCTTCTCAGTAGAGCTGGGCACTGAACTTCTCTGGGTA GCCGAAGTCTCCTTTTCTCCACCTGGGGTAGAGCTTGCATGACCAGAACCCGTAACAAC TGTTGCGGGTTTAGGGGCTGTGGTAGCTGTAAGAAGTTAAAGTCATAGGGTTGG	present (92% cov)	mucin-1 isoform 19 precursor
7,216,105	GACTTCTGCCAGCTCGCTTCTGCTCTGCTGATGGCCTCATCCTGCCACTGTGGCTTTTCA GGCTCTTCCTCCTCTTGCCCTGGCGGACGTGGGGCCCCACTCTGGCTTTCTTCTTTGTA CCAGGCCCTCGCAATGTATTCTTGCTGCTTGTAGGAGAAGCCAGCTTCTTGGAGGAGCC TTTCTCAGACAAACCCTGGGCTGGGCCAGAAGCTAAGGTTGCACTGGAAGATTTTCTAG CAGCTTTCTCTGATTGTTCCTTTAGCAAGTCCAAGACTGTCTCTGAGAAATCAGTATTTATTT	present	HHIP-like protein 2 precursor
7,225,305	CCTGTGATTATCCGAGTTCTAGTAAGCAGAAATCAAACAACTCTGTACATTTGTTACCTG CTTCATCTTCAGCAAAGGAGATGATGAACTTGCTATGGGTGCTGATCAGCCCTGGGAAG GCACAGGACGTGGTGCTGCCCAGGCAAAGATCCTGCTTCTTCCATTTCTTGTTTTTTCTA TCTTCCTGCAAAGCCATAAGTCGACTAGACAAAAAATAAACCCTTATGTTTAGGAATCCAT ATATCCACTCTGCAGAATACTTTTTCTCCTAGAATCAGAGATCCCTGAGTACTAGGACTG	present	HHIP-like protein 2 precursor
7,711,805	CAGCACTCTGCACCTCCCAACTGCAGGTAGAAGTACATCTGGGTGCCACACCCAAGG ACCGAGATGGTCTTGTCTTTGGCCAGCTGGTTCACCAGCATTTTGGGGACAGTGACAG AAATATATGTCAAGTCTATGAGTGAGAGCTGGTTTATAAAGAAGTACATGGGAGTATGCAG AGAGGAGTCAATGTGGATCAGAAGTATCAATGTAATATTCCAAGACACAGCCATCAAAAA TATACTGAAGATAAGCAAGCAGAGGCGGCCAGGGT	missing (73% ident homolog)	olfactory receptor 2T10

*From the online version of Figure 7, 300 bp regions of human CDS regions with no alignment to chimpanzee sequence were snipped out (Column 2). These were searched for in the GenBank nr database, restricted to Hominidae, and showing putative human-specific exons on Chr1.*

### Poster Images

**FIGURE 8:**
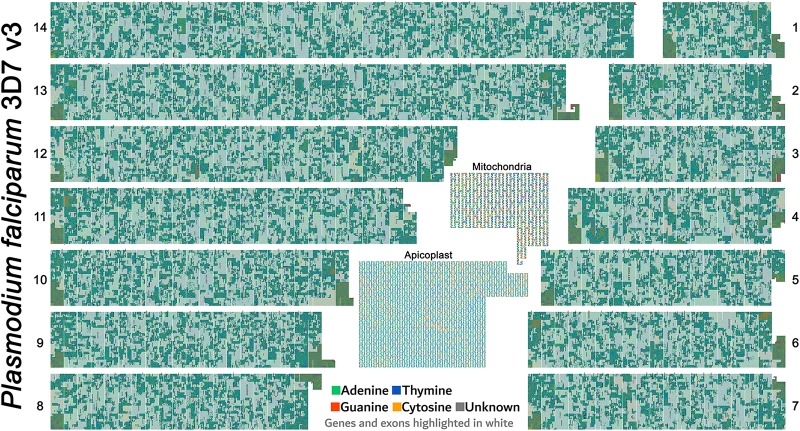
Poster made using FluentDNA outputs. The entire malaria genome was rendered as a poster by arranging the 14 chromosomes in descending order of size. The legend uses organelle genomes to demonstrate the shape of the Peano curve by rendering at scale = 2 with whitespace and magnified. Rendered at standard scale, organelles would be tiny and indiscernibly covered in gene annotations. Major visible features include repetitive telomeres at the end of every chromosome. There are no obvious centromeres. Malaria is visibly much more gene dense than *Anopheles gambiae* or *Homo sapiens*. While Mitochondria are familiar to every geneticist, the Apicoplast organelle is specific to Phylum Apicomplexa protozoan parasites (Gardner et al., 1991; Egea and Lang-Unnasch, 1996).

FluentDNA images are useful for communication of genomic data. For example, Figure 8 shows a poster made displaying the entire malaria genome. In which images made using FluentDNA were arranged using desktop publishing software. The legend uses organelle genomes to demonstrate the shape of the Peano curve.

### Museum Display

The first FluentDNA display was set up in the visitor area of the Millenium Seed Bank as part of *Surviving or Thriving: An exhibition on plants and us* (March 2019–October 2020). *Arabidopsis thaliana* was selected as the display organism because it is well annotated and has a small genome size. The poster (see Supplementary Figure S2) acts as a macro navigation device while the monitor displays the GO Slim functional annotation of the gene as well as the sequence at the position selected (Swarbreck et al., 2008). Using the museum display, it is possible to locate a mitochondrial integration in the centromere of chromosome 2. By touching the visibly orange region (G/C rich) and dragging their finger around, visitors can see genes labeled “mitochondria,” “ATP synthesis,” “Transmembrane electron transport,” etc., Even without detailed knowledge of the technical terms used, every visitor may take away something learned from the display, from the basics of genetic code up to finding clusters of transfer RNA genes. Instructions for creating similar museum displays can be found in Supplementary Data Sheet S2.

## Conclusion

Previous software tools have focused almost exclusively on rendering annotations and markers while the bare sequence is only visible at the smallest scales. We note that FluentDNA is not intended to replace standard genome browsers, but is a useful complement for quality assurance and genome comparison. FluentDNA places emphasis on nucleotides, while placing less emphasis on annotation direction and exon boundaries. Visualization of bare sequence can be informative because gene elements often introduce visible changes in k-mer usage. This is useful in genome assembly for quickly spotting artifacts. FluentDNA is a significant improvement on other direct sequence visualizations (e.g., DDV, DNASkittle) because it can handle multipart FASTA files and scale to viewing entire genomes at once. It also offers a range of capabilities for browsing annotations, protein families and aligned genomes. As a new tool, it does not support every possible file format but extensions are planned, including a VCF render already in development. Finally, FluentDNA allows the creation of posters and museum displays that can make genetic information more accessible to scientists and museum visitors alike.

## Data Availability Statement

The FluentDNA software is available for download at https://github.com/josiahseaman/FluentDNA/releases under the Apache 2.0 open source license. The genome data and visualizations in this MS are available at https://FluentDNA.com/.

## Author Contributions

JS conceived and developed FluentDNA. JS and RB wrote the manuscript.

## Conflict of Interest

The authors declare that the research was conducted in the absence of any commercial or financial relationships that could be construed as a potential conflict of interest.

## Footnotes

1 https://sites.google.com/site/xxcantorxdustxx/

2 https://www.ncbi.nlm.nih.gov/gene/107970260

## Supplementary Material

FIGURE S1 Alignment of chromosome 18 in human (Hg38) and chimpanzee (PanTro6).

FIGURE S2 Museum display for “Surviving or thriving” exhibition.

TABLE S1 Statistics of human (Hg38) versus chimpanzee (PanTro6) whole genome alignment.

DATA SHEET S1 Files referenced in Figure 1 UML diagram.

DATA SHEET S2 Instructions for FluentDNA museum display.

DATA SHEET S3 Run scripts for paper figures.

DATA SHEET S4 Manual annotations of human centromeres.
