# Erratum: FluentDNA: Nucleotide Visualization of Whole Genomes, Annotations, and Alignments

**DOI:** 10.3389/fgene.2020.00745

**Published:** 2020-07-08

**Authors:** Frontiers Production Office

**Affiliations:** Frontiers Media SA, Lausanne, Switzerland

**Keywords:** data visualization, nucleotide visualization, genome assembly, genome browser, chromosome structural variants, genome alignment, comparative genomics, space filling curves

Due to a production error, there was an error in affiliation 1. Instead of “Richmond, VA, United Kingdom,” it should be “Richmond, United Kingdom.”

The publisher apologizes for this mistake. The original article has been updated.

