# Supplementary figures and images for: FluentDNA: Nucleotide Visualization of Whole Genomes, Annotations, and Alignments
Source: Front Genet. 2020 Apr 30;11:292. doi: 10.3389/fgene.2020.00292 (PMC7203487; doi:10.3389/fgene.2020.00292)

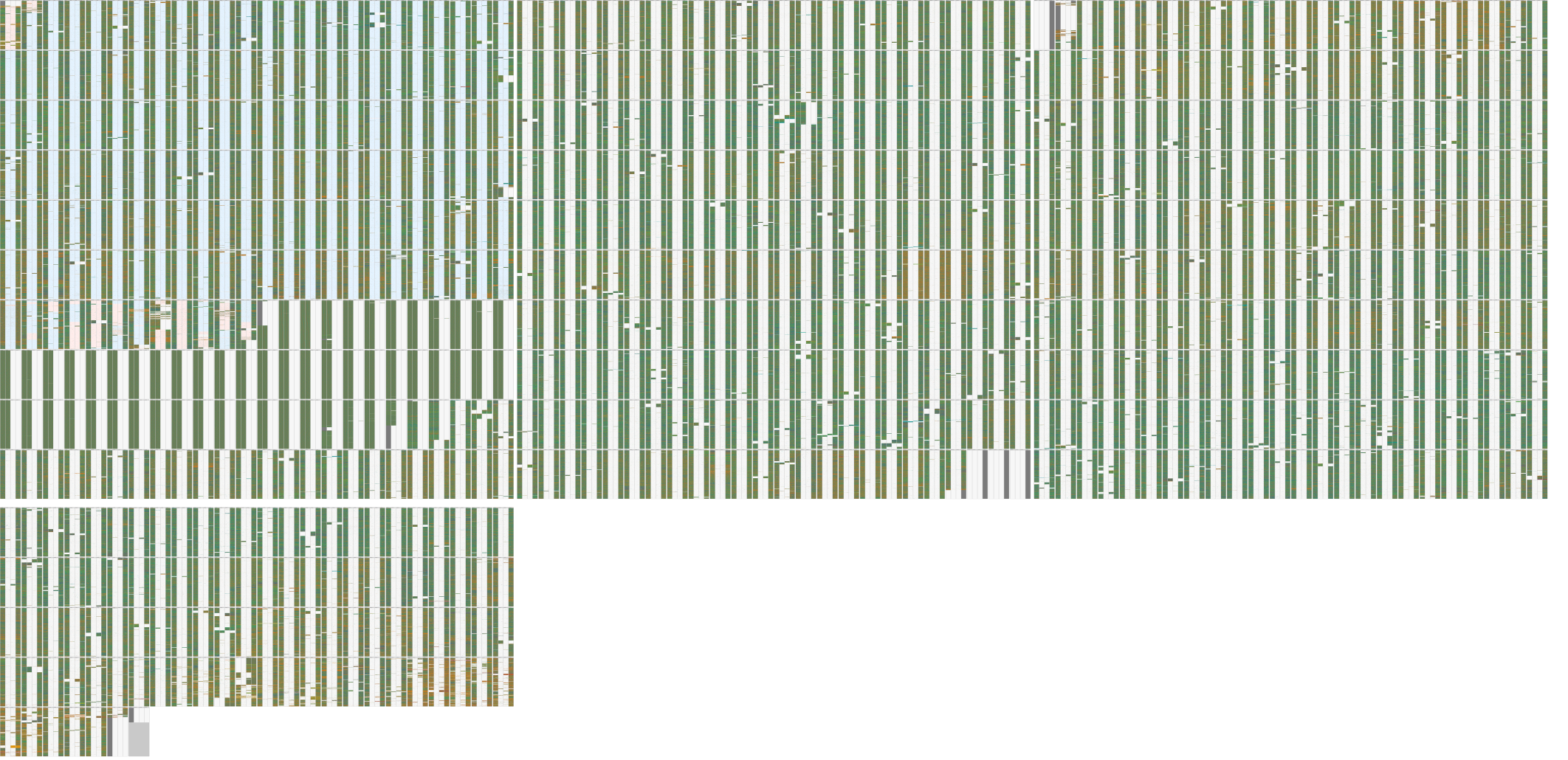

Supplement: FIGURE S1 — Alignment of chromosome 18 in human (Hg38) and chimpanzee (PanTro6). [file Image_1.tiff]

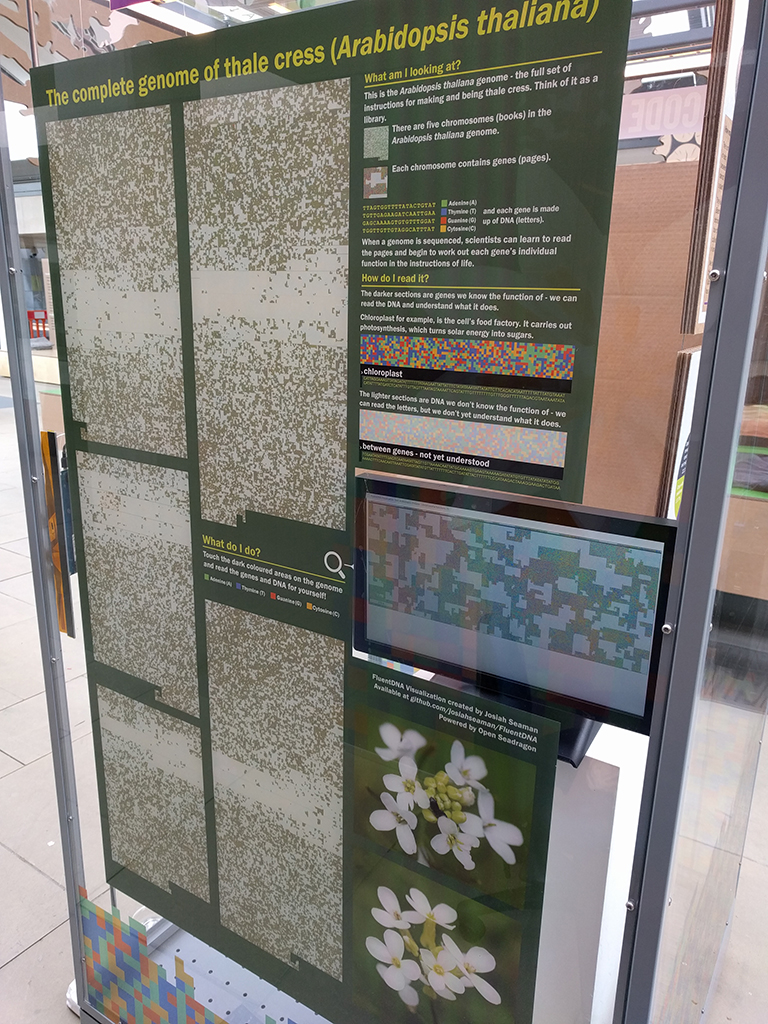

Supplement: FIGURE S2 — Museum display for “Surviving or thriving” exhibition. [file Image_2.jpg]
