# Supplementary material for: FluentDNA: Nucleotide Visualization of Whole Genomes, Annotations, and Alignments
Source: Front Genet. 2020 Apr 30;11:292. doi: 10.3389/fgene.2020.00292 (PMC7203487; doi:10.3389/fgene.2020.00292)
Supplement: DATA SHEET S1 — Files referenced in Figure 1 UML diagram. [file Data_Sheet_1.docx]

Data Sheet 1

# Terms and Files Referenced

**DDVUtils**.py - copy, fetch, filter, sort long lists of contigs​

**Fluentdna**.py - server architecture, rendering modes, table of user input options​

**Layouts**.py - modular layout design enables mouseover sequence for any layout style, user defined layouts​

**TileLayout**.py - Standard rectangular layout visualization. Default is Powers of 10 Layout (Figs. 2, 4 & 5).​ **Index.html** - what is packaged inside a FluentDNA visualization. Contig Spacing JSON, layout JSON.​ nucleotideNumber.js - Inverse algorithm for each layout, chromosome sequence streaming

**Annotations**.py - Read Annotation GFF2 and GFF3. Filtering annotation types. Generate pseudosequence from​ a GFF.

**HighlightedAnnotation**.py - Uses shading in alpha channel to highlight regions covered by an annotation.​ Labels lay over top the sequence and scale by the area available. Larger annotations get bigger labels (Figs. 4 & 5).

**AnnotatedTrackLayout**.py - More traditional rectangles with gene name labels to run alongside sequence​ visualization (Fig. 3 & 8).

**MultipleAlignmentLayout.py** - Proteome MSA gallery. Each Fasta file gets one MSA and one block of​ visualization. MSA blocks are arranged in rows and laid out in 2D according to size (Fig. 6).

**ParallelGenomeLayout**.py - Handles the interlacing of multiple files for Annotation Tracks or Whole Genome​ Alignments. Renders boxes for columns of aligned genome pairs. Whole Genome Alignments are rendered from pseudosequences produced by ChainParser.py (Figs. 3, 7 & 8).

**ChainFiles**.py - parser for UCSC Chained LiftOver files. A Chain contiguous alignment represented by a series​ of Chain entries which each have a size, gap_query, and gap_ref.

**ChainParser**.py - Handles the main logic of parsing whole genome alignments into a visualization. It generates​ two gapped sequences from reference and query genomes, then computes the differences between the two genomes (Figs. 3, 7 & 8).

**AnnotatedAlignment**.py - Apply gaps to GFF pseudosequence as a LiftOver visualization (Fig. 7).​

**Span**.py - Utility class for intersecting ranges of coordinates and handling gaps inside them.​

**UniqueOnlyChainParser**.py - See Unique sequence content by subtracting one genome from another.​ **Ideogram**.py - Peano curve layout designed to look like a packed chromosome under the microscope. This​ layout preserves locality, causing gene regions to appear as bumpy regions like counties on a map (Figs. 2B & 4B).

# Processing Scripts

**Image_resize_script**.py - Set the level of magnification for any image. Useful for generating figures upscaled​ larger than screen display size. Downsample Genome posters for printing.

**Stats_Aggregator**.ipynb - Collect stats on a whole genome alignment across many chromosomes.​

**RepeatAnnotations**.py - Fetch all sequences from RepeatMasker output - Show Repeat Diversity within​ Human

**AnnotationAlignment**.py - Use chain file to perform RepeatAnnotations fetch on a query genome​

**TransposonLayout**.py - Layout for RepeatAnnotations​
