# Supplementary material for: FluentDNA: Nucleotide Visualization of Whole Genomes, Annotations, and Alignments
Source: Front Genet. 2020 Apr 30;11:292. doi: 10.3389/fgene.2020.00292 (PMC7203487; doi:10.3389/fgene.2020.00292)
Supplement: DATA SHEET S2 — Instructions for FluentDNA museum display. [file Data_Sheet_2.docx]

Data Sheet 2

FluentDNA museum display

These instructions step through everything required to setup a FluentDNA museum display. The original image file generated by FluentDNA was edited in Photoshop to add the interpretation text and legend and then reprocessed by DeepZoom to ensure the monitor and poster match. Finally, since gene labels are not rendered on the poster, the color palette was modified so that genes were saturated colors and intergenic regions are lightened to draw attention to the genes.

The *Arabidopsis thaliana* ​​genome required special processing for this exhibit. Chromosomes were broken into a series of genes with the FASTA entry name carrying the functional annotation for that gene. This was used as a specialized annotation retrieval since FluentDNA shows the name of each FASTA entry under the mouse. GO Slim was selected to minimize the length of technical jargon in the annotation. Centromeres, largely lacking genes, are easily visible in the final display.

The physical setup was a 2 meter tall display case with a 1.5 meter poster behind plexiglass. Behind the poster was a 72cm x 124cm touch sensitive film manufactured by Displax. Since touch sensors use changes in electrostatics, they can sense touch through several layers of material. The Displax touch sensor sends touch events to a computer running FluentDNA with a copy of the exact same poster. The monitor positioned inside the poster shows a magnified version of the poster (Figure S2).

First, sequence and functional annotation were downloaded from TAIR10

[(https://www.arabidopsis.org/download/index-auto.jsp?dir=%2Fdownload_files%2FGO_and_PO_Annotation](https://www.arabidopsis.org/download/index-auto.jsp?dir=%2Fdownload_files%2FGO_and_PO_Annotations%2FGene_Ontology_Annotations)​ [s %2FGene_Ontology_Annotations](https://www.arabidopsis.org/download/index-auto.jsp?dir=%2Fdownload_files%2FGO_and_PO_Annotations%2FGene_Ontology_Annotations))​ (accessed March 2019). GO Slim was selected to minimize the length of technical jargon in the annotation. The annotation was processed through

Functional_Annotation_Arabidopsis.ipynb to remove all genes that did not have a known function. The remaining gene annotations had their numeric names replaced with their GO Slim function and placed in a new GFF file.

The function **write_functional_gene_contigs_from_chromosome()**​ reads nucleotide sequence​ and gene annotation together and outputs consecutive contigs with the fasta header as the function followed by the complete sequence inside the gene annotation. Regions with no annotation are all labeled “between genes - not yet understood”. Each FASTA file then contains one chromosome with a FASTA entry for each gene and intergenic region. There are some obvious downsides to this approach, primarily that overlapping genes compete for label space. However, this was a quick and effective way to get annotation mouseover in FluentDNA and more than accurate enough for a museum display.

Using the modified FASTA file, FluentDNA can be used to render a poster of one chromosome. In order to layout multiple chromosomes using the Ideogram layout, a modified version of the software must be used. The “wakehurst” branch on GitHub contains some special exceptions that must be modified for each project. The origin coordinates of each of the chromosomes are calculated and entered by hand to whatever poster layout the developer prefers. While it appears the poster uses HighlightedAnnotations.py it actually uses the FASTA headers themselves. Whenever a contig called “between genes - not yet understood” is rendered, the color palette is switched to a lighter shade which mimics highlighting. This allows attention to be drawn to the genes rendered in saturated dark colors while the intergenic regions are whitened out. Gene labels are not rendered since the monitor already serves that function.

After the initial render, the now 150MB PNG file was brought into Photoshop where the rest of the poster design was carried out. Poster layout design was done by Samantha Seaman. Final color choice and fonts were done by Adomas Mockus at Rockbrook Engineering. The final poster design was re-rendered back into FluentDNA DeepZoom stack so that the monitor and poster matched perfectly.

When a user touches a point on the poster, Displax Connect interprets that into a x,y coordinate on the touch surface. This is sent through Windows HID service to a screen. The display screen is purely informative and doesn’t match the User Interface represented by a poster. Therefore, a second logical screen was required to act as a digital counterpart to the poster. On HDMI and newer displays Windows logical screens always map to a physical device. However, Windows contains legacy support for VGA monitors that would not report their resolution. Declaring a monitor as a VGA monitor in Windows allows one to add another monitor in display settings and manually set the resolution without Windows requiring feedback from a physical monitor.

The small desktop used for the display did not have an onboard VGA port, so it was necessary to plug in a USB to VGA adapter. Bridging VGA pins 1 (Red) and 6 (Red Ground) with an insulated cable caused the computer to recognize it as a connected monitor of unknown resolution. The resolution was then set to exactly match the resolution of the Displax touch surface and place in Portrait orientation.

The faked logical monitor is then used to translate Displax touch coordinates into HTML coordinates for a portion of the webpage that generates JavaScript commands but is never seen by the user. The OpenSeadragon Navigator element is placed on this second screen since it never changes position and allows the user to navigate globally. In each display, the positioning of the navigator on the second screen will require manual adjustment to match the poster position and scale. This process was helped greatly by using TeamViewer on a laptop to see the contents of the virtual screen and Chrome’s ability to live edit JavaScript source files.

Finally, in order to get the sequence to display, a mouseover event must be simulated in the middle of the screen. A quarter-second loop which creates a fake mouse hover event and asserts the zoom level was enough to trigger FluentDNA mouseover functionality. The whole setup is shut down at night and boots up every morning using a BIOS rule that checks for power. The program LaunchLater

[(https://jeffcox111.github.io/LaunchLater](https://jeffcox111.github.io/LaunchLater/)​ [/)](https://jeffcox111.github.io/LaunchLater/)​ is used to launch the FluentDNA server, then a browser 5 minutes later. The Chrome browser is run in Kiosk mode which removes all the usual browser decorations and pointed directly to the locally served webpage. Chrome windows will preserve their size after reboots and can span multiple screens as long as they are not Maximized. Using this setup one browser window handles both the display and hidden UI elements.

***Figure S2 Legend:*​** *Final poster displayed in the Millenium Seed Bank. The left two thirds of the poster are backed by* *Displax touch sensitive film (visible as an orange strip on the far left) for user input. On the right, an embedded computer monitor running FluentDNA with a digital copy of the poster. Touching the poster causes the monitor to zoom in on the corresponding place in the genome and display the sequence and function of the gene that was touched.*
