# Supplementary material for: FluentDNA: Nucleotide Visualization of Whole Genomes, Annotations, and Alignments
Source: Front Genet. 2020 Apr 30;11:292. doi: 10.3389/fgene.2020.00292 (PMC7203487; doi:10.3389/fgene.2020.00292)
Supplement: DATA SHEET S3 — Run scripts for paper figures. [file Data_Sheet_3.docx]

## # Figure 4A

./FluentDNA --fasta="D:\Genomes\Human\hg38_chr18.fa" --ref_annotation="D:\Genomes\Human\gencode.v30.annotation.gff3" --sort_contigs --outname="Human Genome Hg38 chr18 with Gencode v30" --contigs chr18 --no_titles --custom_layout="([100, 405, 65, 50],[0, 0, 1, 3])"

## # Figure 4B

./FluentDNA --fasta="D:\Genomes\Human\hg38.fa" --ref_annotation="D:\Genomes\Human\gencode.v30.annotation.gff3" --sort_contigs --outname="Human Ideogram Hg38 chr18 with Gencode v30" --contigs chr18 --radix="([3,3,3,3,3, 27], [5,3,3,3,3,3,53],1,1)" --no_titles

Nucleotides per mega row:
Tile Layout: 100*405*65 = 2,632,500
Ideogram: 3*3*3*3*3*27 * 5*3*3*3*3 = 2657205

## #Figure 5: Whole Human Genome with Annotation

./FluentDNA --fasta="/science/projects/buggs_lab/josiah/Human/hg38.fa" --ref_annotation="/science/projects/buggs_lab/josiah/Human/gencode.v30.annotation.gff3" --outname="Human Hg38 Chromosomes and Gencodev30 Genes" --contigs chr1 chr2 chr3 chr4 chr5 chr6 chr7 chr8 chr9 chr10 chr11 chr12 chr13 chr14 chr15 chr16 chr17 chr18 chr19 chr20 chr21 chr22 chrX chrY chrM

## #Fig. 6: MSA Gallery of Chr19 Repeats

### # Generate MSA files using script to parse RepeatMasker csv

python scripts/RepeatMaskerParse.py supplemental\RepeatMasker_all_alignment.csv "D:\Genomes\Human\hg38_chr18.fa" supplemental\Hg38_chr18_repeats_length_sorted chr18

### # Render MSA Gallery from sequence folder

./FluentDNA --layout=alignment --fasta="supplemental\Hg38_chr18_repeats_length_sorted" --outname="Human Hg38 Chromosome 18 Repeats - alphabetical"

### # Figure S2: Human/Chimp Whole Genome Alignment

./FluentDNA --fasta="D:\Genomes\Human\Hg38.fa" --extrafastas "D:\Genomes\Chimpanzee\panTro6.fa" --chainfile="D:\Genomes\Human\hg38ToPanTro6.over.chain" --outname="Human Hg38 vs Chimpanzee PanTro6" --sort_contigs --contigs chr1 chr2 chr3 chr4 chr5 chr6 chr7 chr8 chr9 chr10 chr11 chr12 chr13 chr14 chr15 chr16 chr17 chr18 chr19 chr20 chr21 chr22 chrX chrY

./FluentDNA --fasta="D:\Genomes\Human\chroms\chr18.fa" --extrafastas "D:\Genomes\Chimpanzee\panTro6.fa" --chainfile="D:\Genomes\Human\hg38ToPanTro6.over.chain" --outname="Human Hg38 chr18 vs Chimpanzee PanTro6" --contigs chr18

## #Figure 7: Annotating centromeres and Unique Human Genes

Multiple commands were required to prep this analysis.

### # Human Unique Sequence with N's

fluentdna.py --fasta=D:\Genomes\Human\Hg38.fa --chainfile=D:\Genomes\Human\hg38ToPanTro6.over.chain "--outname=Unique Human Hg38 vs Chimpanzee PanTro6" --preserve_Ns --contigs chr1 chr2 chr3 chr4 chr5 chr6 chr7 chr8 chr9 chr10 chr11 chr12 chr13 chr14 chr15 chr16 chr17 chr18 chr19 chr20 chr21 chr22 chrX chrY --layout=unique

### # Figure 7A: Output only Human Sequence unaligned to PanTro6

Human Unique Sequence (Layout Tweaked)

fluentdna.py --fasta="D:\Projects\FluentDNA\DDV\www-data\dnadata\Unique Human Hg38 vs Chimpanzee PanTro6_\sources\Unique Human Hg38 vs Chimpanzee PanTro6___134760677bp.fa" --outname="Unique Human vs Chimp PanTro6" --custom_layout="([100, 100* 10, 102, 10, 3, 4, 999], [0, 0, 6, 6 * 3, 6 * (3 ** 2), 6 * (3 ** 3), 6 * (3 ** 4)])"

### #Fig 7B: Generate annotation pseudo sequence

./FluentDNA --fasta="D:\Genomes\Human\hg38.fa" --ref_annotation="D:\Genomes\Human\gencode.v30.annotation.gff3" --sort_contigs --outname="Human Genome Hg38 with Gencode v30" --layout=annotation_track --annotation_width=100

**Note:** Use text editor to strip off scaffold names *_HG38_unique and *_gencode_unique

### # Use pseudo sequence as input to Unique Layout

./FluentDNA --fasta="D:\Projects\FluentDNA-2.4.1\www-data\dnadata\Human Genome Hg38 with Gencode v30\gencode.v30.annotation.gff3.fa" --outname="Human Specific Gene Annotations Gencode v30 vs PanTro6" --layout=unique --chainfile="D:\Genomes\Human\hg38ToPanTro6.over.chain" --custom_layout="([100, 100* 10, 102, 10, 3, 4, 999], [0, 0, 6, 6 * 3, 6 * (3 ** 2), 6 * (3 ** 3), 6 * (3 ** 4)])" --contigs chr1 chr2 chr3 chr4 chr5 chr6 chr7 chr8 chr9 chr10 chr11 chr12 chr13 chr14 chr15 chr16 chr17 chr18 chr19 chr20 chr21 chr22 chrX chrY

### # Pseudo sequence for Centromere annotation

./FluentDNA --outname="Manual Centromere Locations" --fasta="D:\Projects\FluentDNA-2.4.1\www-data\dnadata\Unique Human vs Chimp PanTro6\sources\Unique Human Hg38 vs Chimpanzee PanTro6___134760677bp.fa" --ref_annotation="D:\Genomes\Human\Unique Human Centromere Locations.gff3" --layout=annotation_track --annotation_width=100 --custom_layout="([100, 100* 10, 102, 10, 3, 4, 999], [0, 0, 6, 6 * 3, 6 * (3 ** 2), 6 * (3 ** 3), 6 * (3 ** 4)])" --contigs chr1 chr2 chr3 chr4 chr5 chr6 chr7 chr8 chr9 chr10 chr11 chr12 chr13 chr14 chr15 chr16 chr17 chr18 chr19 chr20 chr21 chr22 chrX chrY

### # Render Human Unique Genes and Sequence Together

./FluentDNA --fasta="D:\Projects\FluentDNA-2.4.1\www-data\dnadata\Human Genome Hg38 with Gencode v30\sources" --extrafastas "D:\Projects\FluentDNA-2.4.1\www-data\dnadata\Unique Human vs Chimp PanTro6\sources\Unique Human Hg38 vs Chimpanzee PanTro6___134760677bp.fa" --outname="Human Unique Genes vs PanTro6 (Gencode v30)" --layout=parallel --contigs chr1 chr2 chr3 chr4 chr5 chr6 chr7 chr8 chr9 chr10 chr11 chr12 chr13 chr14 chr15 chr16 chr17 chr18 chr19 chr20 chr21 chr22 chrX chrY

### # Testing annotation spacing with Unique

Manual Annotation Track

./FluentDNA --outname="Manual Annotation Test chr21" --fasta="D:\Projects\FluentDNA-2.4.1\www-data\dnadata\Human Genome Hg38 with Gencode v30\gencode.v30.annotation.gff3.fa" --extra_fastas "D:\Genomes\Human\chroms\chr21.fa" --contigs chr21

## # Supplemental: Whole Genome alignment for Hg38 and PanTro6 - all chromosomes

FluentDNA.exe --fasta="D:\josiah\Projects\DDV\FluentDNA\results\Human Hg38 vs Chimpanzee PanTro6_chr1\sources\chr1_hg38_gapped.fa" --extrafastas "D:\josiah\Projects\DDV\FluentDNA\results\Human Hg38 vs Chimpanzee PanTro6_chr1\sources\chr1_hg38_unique.fa" "D:\josiah\Projects\DDV\FluentDNA\results\Human Hg38 vs Chimpanzee PanTro6_chr1\sources\panTro6_to_hg38_chr1_unique.fa" "D:\josiah\Projects\DDV\FluentDNA\results\Human Hg38 vs Chimpanzee PanTro6_chr1\sources\panTro6_to_hg38_chr1_gapped.fa" --outname="Human Hg38 vs Chimpanzee PanTro6_chr1"

FluentDNA.exe --fasta="D:\josiah\Projects\DDV\FluentDNA\results\Human Hg38 vs Chimpanzee PanTro6_chr2\sources\chr2_hg38_gapped.fa" --extrafastas "D:\josiah\Projects\DDV\FluentDNA\results\Human Hg38 vs Chimpanzee PanTro6_chr2\sources\chr2_hg38_unique.fa" "D:\josiah\Projects\DDV\FluentDNA\results\Human Hg38 vs Chimpanzee PanTro6_chr2\sources\panTro6_to_hg38_chr2_unique.fa" "D:\josiah\Projects\DDV\FluentDNA\results\Human Hg38 vs Chimpanzee PanTro6_chr2\sources\panTro6_to_hg38_chr2_gapped.fa" --outname="Human Hg38 vs Chimpanzee PanTro6_chr2"

FluentDNA.exe --fasta="D:\josiah\Projects\DDV\FluentDNA\results\Human Hg38 vs Chimpanzee PanTro6_chr3\sources\chr3_hg38_gapped.fa" --extrafastas "D:\josiah\Projects\DDV\FluentDNA\results\Human Hg38 vs Chimpanzee PanTro6_chr3\sources\chr3_hg38_unique.fa" "D:\josiah\Projects\DDV\FluentDNA\results\Human Hg38 vs Chimpanzee PanTro6_chr3\sources\panTro6_to_hg38_chr3_unique.fa" "D:\josiah\Projects\DDV\FluentDNA\results\Human Hg38 vs Chimpanzee PanTro6_chr3\sources\panTro6_to_hg38_chr3_gapped.fa" --outname="Human Hg38 vs Chimpanzee PanTro6_chr3"

FluentDNA.exe --fasta="D:\josiah\Projects\DDV\FluentDNA\results\Human Hg38 vs Chimpanzee PanTro6_chr4\sources\chr4_hg38_gapped.fa" --extrafastas "D:\josiah\Projects\DDV\FluentDNA\results\Human Hg38 vs Chimpanzee PanTro6_chr4\sources\chr4_hg38_unique.fa" "D:\josiah\Projects\DDV\FluentDNA\results\Human Hg38 vs Chimpanzee PanTro6_chr4\sources\panTro6_to_hg38_chr4_unique.fa" "D:\josiah\Projects\DDV\FluentDNA\results\Human Hg38 vs Chimpanzee PanTro6_chr4\sources\panTro6_to_hg38_chr4_gapped.fa" --outname="Human Hg38 vs Chimpanzee PanTro6_chr4"

FluentDNA.exe --fasta="D:\josiah\Projects\DDV\FluentDNA\results\Human Hg38 vs Chimpanzee PanTro6_chr5\sources\chr5_hg38_gapped.fa" --extrafastas "D:\josiah\Projects\DDV\FluentDNA\results\Human Hg38 vs Chimpanzee PanTro6_chr5\sources\chr5_hg38_unique.fa" "D:\josiah\Projects\DDV\FluentDNA\results\Human Hg38 vs Chimpanzee PanTro6_chr5\sources\panTro6_to_hg38_chr5_unique.fa" "D:\josiah\Projects\DDV\FluentDNA\results\Human Hg38 vs Chimpanzee PanTro6_chr5\sources\panTro6_to_hg38_chr5_gapped.fa" --outname="Human Hg38 vs Chimpanzee PanTro6_chr5"

FluentDNA.exe --fasta="D:\josiah\Projects\DDV\FluentDNA\results\Human Hg38 vs Chimpanzee PanTro6_chr6\sources\chr6_hg38_gapped.fa" --extrafastas "D:\josiah\Projects\DDV\FluentDNA\results\Human Hg38 vs Chimpanzee PanTro6_chr6\sources\chr6_hg38_unique.fa" "D:\josiah\Projects\DDV\FluentDNA\results\Human Hg38 vs Chimpanzee PanTro6_chr6\sources\panTro6_to_hg38_chr6_unique.fa" "D:\josiah\Projects\DDV\FluentDNA\results\Human Hg38 vs Chimpanzee PanTro6_chr6\sources\panTro6_to_hg38_chr6_gapped.fa" --outname="Human Hg38 vs Chimpanzee PanTro6_chr6"

FluentDNA.exe --fasta="D:\josiah\Projects\DDV\FluentDNA\results\Human Hg38 vs Chimpanzee PanTro6_chr7\sources\chr7_hg38_gapped.fa" --extrafastas "D:\josiah\Projects\DDV\FluentDNA\results\Human Hg38 vs Chimpanzee PanTro6_chr7\sources\chr7_hg38_unique.fa" "D:\josiah\Projects\DDV\FluentDNA\results\Human Hg38 vs Chimpanzee PanTro6_chr7\sources\panTro6_to_hg38_chr7_unique.fa" "D:\josiah\Projects\DDV\FluentDNA\results\Human Hg38 vs Chimpanzee PanTro6_chr7\sources\panTro6_to_hg38_chr7_gapped.fa" --outname="Human Hg38 vs Chimpanzee PanTro6_chr7"

FluentDNA.exe --fasta="D:\josiah\Projects\DDV\FluentDNA\results\Human Hg38 vs Chimpanzee PanTro6_chr8\sources\chr8_hg38_gapped.fa" --extrafastas "D:\josiah\Projects\DDV\FluentDNA\results\Human Hg38 vs Chimpanzee PanTro6_chr8\sources\chr8_hg38_unique.fa" "D:\josiah\Projects\DDV\FluentDNA\results\Human Hg38 vs Chimpanzee PanTro6_chr8\sources\panTro6_to_hg38_chr8_unique.fa" "D:\josiah\Projects\DDV\FluentDNA\results\Human Hg38 vs Chimpanzee PanTro6_chr8\sources\panTro6_to_hg38_chr8_gapped.fa" --outname="Human Hg38 vs Chimpanzee PanTro6_chr8"

FluentDNA.exe --fasta="D:\josiah\Projects\DDV\FluentDNA\results\Human Hg38 vs Chimpanzee PanTro6_chr9\sources\chr9_hg38_gapped.fa" --extrafastas "D:\josiah\Projects\DDV\FluentDNA\results\Human Hg38 vs Chimpanzee PanTro6_chr9\sources\chr9_hg38_unique.fa" "D:\josiah\Projects\DDV\FluentDNA\results\Human Hg38 vs Chimpanzee PanTro6_chr9\sources\panTro6_to_hg38_chr9_unique.fa" "D:\josiah\Projects\DDV\FluentDNA\results\Human Hg38 vs Chimpanzee PanTro6_chr9\sources\panTro6_to_hg38_chr9_gapped.fa" --outname="Human Hg38 vs Chimpanzee PanTro6_chr9"

FluentDNA.exe --fasta="D:\josiah\Projects\DDV\FluentDNA\results\Human Hg38 vs Chimpanzee PanTro6_chr10\sources\chr10_hg38_gapped.fa" --extrafastas "D:\josiah\Projects\DDV\FluentDNA\results\Human Hg38 vs Chimpanzee PanTro6_chr10\sources\chr10_hg38_unique.fa" "D:\josiah\Projects\DDV\FluentDNA\results\Human Hg38 vs Chimpanzee PanTro6_chr10\sources\panTro6_to_hg38_chr10_unique.fa" "D:\josiah\Projects\DDV\FluentDNA\results\Human Hg38 vs Chimpanzee PanTro6_chr10\sources\panTro6_to_hg38_chr10_gapped.fa" --outname="Human Hg38 vs Chimpanzee PanTro6_chr10"

FluentDNA.exe --fasta="D:\josiah\Projects\DDV\FluentDNA\results\Human Hg38 vs Chimpanzee PanTro6_chr11\sources\chr11_hg38_gapped.fa" --extrafastas "D:\josiah\Projects\DDV\FluentDNA\results\Human Hg38 vs Chimpanzee PanTro6_chr11\sources\chr11_hg38_unique.fa" "D:\josiah\Projects\DDV\FluentDNA\results\Human Hg38 vs Chimpanzee PanTro6_chr11\sources\panTro6_to_hg38_chr11_unique.fa" "D:\josiah\Projects\DDV\FluentDNA\results\Human Hg38 vs Chimpanzee PanTro6_chr11\sources\panTro6_to_hg38_chr11_gapped.fa" --outname="Human Hg38 vs Chimpanzee PanTro6_chr11"

FluentDNA.exe --fasta="D:\josiah\Projects\DDV\FluentDNA\results\Human Hg38 vs Chimpanzee PanTro6_chr12\sources\chr12_hg38_gapped.fa" --extrafastas "D:\josiah\Projects\DDV\FluentDNA\results\Human Hg38 vs Chimpanzee PanTro6_chr12\sources\chr12_hg38_unique.fa" "D:\josiah\Projects\DDV\FluentDNA\results\Human Hg38 vs Chimpanzee PanTro6_chr12\sources\panTro6_to_hg38_chr12_unique.fa" "D:\josiah\Projects\DDV\FluentDNA\results\Human Hg38 vs Chimpanzee PanTro6_chr12\sources\panTro6_to_hg38_chr12_gapped.fa" --outname="Human Hg38 vs Chimpanzee PanTro6_chr12"

FluentDNA.exe --fasta="D:\josiah\Projects\DDV\FluentDNA\results\Human Hg38 vs Chimpanzee PanTro6_chr13\sources\chr13_hg38_gapped.fa" --extrafastas "D:\josiah\Projects\DDV\FluentDNA\results\Human Hg38 vs Chimpanzee PanTro6_chr13\sources\chr13_hg38_unique.fa" "D:\josiah\Projects\DDV\FluentDNA\results\Human Hg38 vs Chimpanzee PanTro6_chr13\sources\panTro6_to_hg38_chr13_unique.fa" "D:\josiah\Projects\DDV\FluentDNA\results\Human Hg38 vs Chimpanzee PanTro6_chr13\sources\panTro6_to_hg38_chr13_gapped.fa" --outname="Human Hg38 vs Chimpanzee PanTro6_chr13"

FluentDNA.exe --fasta="D:\josiah\Projects\DDV\FluentDNA\results\Human Hg38 vs Chimpanzee PanTro6_chr14\sources\chr14_hg38_gapped.fa" --extrafastas "D:\josiah\Projects\DDV\FluentDNA\results\Human Hg38 vs Chimpanzee PanTro6_chr14\sources\chr14_hg38_unique.fa" "D:\josiah\Projects\DDV\FluentDNA\results\Human Hg38 vs Chimpanzee PanTro6_chr14\sources\panTro6_to_hg38_chr14_unique.fa" "D:\josiah\Projects\DDV\FluentDNA\results\Human Hg38 vs Chimpanzee PanTro6_chr14\sources\panTro6_to_hg38_chr14_gapped.fa" --outname="Human Hg38 vs Chimpanzee PanTro6_chr14"

FluentDNA.exe --fasta="D:\josiah\Projects\DDV\FluentDNA\results\Human Hg38 vs Chimpanzee PanTro6_chr15\sources\chr15_hg38_gapped.fa" --extrafastas "D:\josiah\Projects\DDV\FluentDNA\results\Human Hg38 vs Chimpanzee PanTro6_chr15\sources\chr15_hg38_unique.fa" "D:\josiah\Projects\DDV\FluentDNA\results\Human Hg38 vs Chimpanzee PanTro6_chr15\sources\panTro6_to_hg38_chr15_unique.fa" "D:\josiah\Projects\DDV\FluentDNA\results\Human Hg38 vs Chimpanzee PanTro6_chr15\sources\panTro6_to_hg38_chr15_gapped.fa" --outname="Human Hg38 vs Chimpanzee PanTro6_chr15"

FluentDNA.exe --fasta="D:\josiah\Projects\DDV\FluentDNA\results\Human Hg38 vs Chimpanzee PanTro6_chr16\sources\chr16_hg38_gapped.fa" --extrafastas "D:\josiah\Projects\DDV\FluentDNA\results\Human Hg38 vs Chimpanzee PanTro6_chr16\sources\chr16_hg38_unique.fa" "D:\josiah\Projects\DDV\FluentDNA\results\Human Hg38 vs Chimpanzee PanTro6_chr16\sources\panTro6_to_hg38_chr16_unique.fa" "D:\josiah\Projects\DDV\FluentDNA\results\Human Hg38 vs Chimpanzee PanTro6_chr16\sources\panTro6_to_hg38_chr16_gapped.fa" --outname="Human Hg38 vs Chimpanzee PanTro6_chr16"

FluentDNA.exe --fasta="D:\josiah\Projects\DDV\FluentDNA\results\Human Hg38 vs Chimpanzee PanTro6_chr17\sources\chr17_hg38_gapped.fa" --extrafastas "D:\josiah\Projects\DDV\FluentDNA\results\Human Hg38 vs Chimpanzee PanTro6_chr17\sources\chr17_hg38_unique.fa" "D:\josiah\Projects\DDV\FluentDNA\results\Human Hg38 vs Chimpanzee PanTro6_chr17\sources\panTro6_to_hg38_chr17_unique.fa" "D:\josiah\Projects\DDV\FluentDNA\results\Human Hg38 vs Chimpanzee PanTro6_chr17\sources\panTro6_to_hg38_chr17_gapped.fa" --outname="Human Hg38 vs Chimpanzee PanTro6_chr17"

FluentDNA.exe --fasta="D:\josiah\Projects\DDV\FluentDNA\results\Human Hg38 vs Chimpanzee PanTro6_chr18\sources\chr18_hg38_gapped.fa" --extrafastas "D:\josiah\Projects\DDV\FluentDNA\results\Human Hg38 vs Chimpanzee PanTro6_chr18\sources\chr18_hg38_unique.fa" "D:\josiah\Projects\DDV\FluentDNA\results\Human Hg38 vs Chimpanzee PanTro6_chr18\sources\panTro6_to_hg38_chr18_unique.fa" "D:\josiah\Projects\DDV\FluentDNA\results\Human Hg38 vs Chimpanzee PanTro6_chr18\sources\panTro6_to_hg38_chr18_gapped.fa" --outname="Human Hg38 vs Chimpanzee PanTro6_chr18"

FluentDNA.exe --fasta="D:\josiah\Projects\DDV\FluentDNA\results\Human Hg38 vs Chimpanzee PanTro6_chr19\sources\chr19_hg38_gapped.fa" --extrafastas "D:\josiah\Projects\DDV\FluentDNA\results\Human Hg38 vs Chimpanzee PanTro6_chr19\sources\chr19_hg38_unique.fa" "D:\josiah\Projects\DDV\FluentDNA\results\Human Hg38 vs Chimpanzee PanTro6_chr19\sources\panTro6_to_hg38_chr19_unique.fa" "D:\josiah\Projects\DDV\FluentDNA\results\Human Hg38 vs Chimpanzee PanTro6_chr19\sources\panTro6_to_hg38_chr19_gapped.fa" --outname="Human Hg38 vs Chimpanzee PanTro6_chr19"

FluentDNA.exe --fasta="D:\josiah\Projects\DDV\FluentDNA\results\Human Hg38 vs Chimpanzee PanTro6_chr20\sources\chr20_hg38_gapped.fa" --extrafastas "D:\josiah\Projects\DDV\FluentDNA\results\Human Hg38 vs Chimpanzee PanTro6_chr20\sources\chr20_hg38_unique.fa" "D:\josiah\Projects\DDV\FluentDNA\results\Human Hg38 vs Chimpanzee PanTro6_chr20\sources\panTro6_to_hg38_chr20_unique.fa" "D:\josiah\Projects\DDV\FluentDNA\results\Human Hg38 vs Chimpanzee PanTro6_chr20\sources\panTro6_to_hg38_chr20_gapped.fa" --outname="Human Hg38 vs Chimpanzee PanTro6_chr20"

FluentDNA.exe --fasta="D:\josiah\Projects\DDV\FluentDNA\results\Human Hg38 vs Chimpanzee PanTro6_chr21\sources\chr21_hg38_gapped.fa" --extrafastas "D:\josiah\Projects\DDV\FluentDNA\results\Human Hg38 vs Chimpanzee PanTro6_chr21\sources\chr21_hg38_unique.fa" "D:\josiah\Projects\DDV\FluentDNA\results\Human Hg38 vs Chimpanzee PanTro6_chr21\sources\panTro6_to_hg38_chr21_unique.fa" "D:\josiah\Projects\DDV\FluentDNA\results\Human Hg38 vs Chimpanzee PanTro6_chr21\sources\panTro6_to_hg38_chr21_gapped.fa" --outname="Human Hg38 vs Chimpanzee PanTro6_chr21"

FluentDNA.exe --fasta="D:\josiah\Projects\DDV\FluentDNA\results\Human Hg38 vs Chimpanzee PanTro6_chr22\sources\chr22_hg38_gapped.fa" --extrafastas "D:\josiah\Projects\DDV\FluentDNA\results\Human Hg38 vs Chimpanzee PanTro6_chr22\sources\chr22_hg38_unique.fa" "D:\josiah\Projects\DDV\FluentDNA\results\Human Hg38 vs Chimpanzee PanTro6_chr22\sources\panTro6_to_hg38_chr22_unique.fa" "D:\josiah\Projects\DDV\FluentDNA\results\Human Hg38 vs Chimpanzee PanTro6_chr22\sources\panTro6_to_hg38_chr22_gapped.fa" --outname="Human Hg38 vs Chimpanzee PanTro6_chr22"

FluentDNA.exe --fasta="D:\josiah\Projects\DDV\FluentDNA\results\Human Hg38 vs Chimpanzee PanTro6_chrX\sources\chrX_hg38_gapped.fa" --extrafastas "D:\josiah\Projects\DDV\FluentDNA\results\Human Hg38 vs Chimpanzee PanTro6_chrX\sources\chrX_hg38_unique.fa" "D:\josiah\Projects\DDV\FluentDNA\results\Human Hg38 vs Chimpanzee PanTro6_chrX\sources\panTro6_to_hg38_chrX_unique.fa" "D:\josiah\Projects\DDV\FluentDNA\results\Human Hg38 vs Chimpanzee PanTro6_chrX\sources\panTro6_to_hg38_chrX_gapped.fa" --outname="Human Hg38 vs Chimpanzee PanTro6_chrX"

FluentDNA.exe --fasta="D:\josiah\Projects\DDV\FluentDNA\results\Human Hg38 vs Chimpanzee PanTro6_chrY\sources\chrY_hg38_gapped.fa" --extrafastas "D:\josiah\Projects\DDV\FluentDNA\results\Human Hg38 vs Chimpanzee PanTro6_chrY\sources\chrY_hg38_unique.fa" "D:\josiah\Projects\DDV\FluentDNA\results\Human Hg38 vs Chimpanzee PanTro6_chrY\sources\panTro6_to_hg38_chrY_unique.fa" "D:\josiah\Projects\DDV\FluentDNA\results\Human Hg38 vs Chimpanzee PanTro6_chrY\sources\panTro6_to_hg38_chrY_gapped.fa" --outname="Human Hg38 vs Chimpanzee PanTro6_chrY"
